# COVID-19 Infection in Children, Infants and Pregnant Subjects: An Overview of Recent Insights and Therapies

**DOI:** 10.3390/microorganisms9091964

**Published:** 2021-09-16

**Authors:** Giuseppina Malcangi, Alessio Danilo Inchingolo, Angelo Michele Inchingolo, Luigi Santacroce, Grazia Marinelli, Antonio Mancini, Luigi Vimercati, Maria Elena Maggiore, Maria Teresa D’Oria, Denisa Hazballa, Ioana Roxana Bordea, Edit Xhajanka, Antonio Scarano, Marco Farronato, Gianluca Martino Tartaglia, Delia Giovanniello, Ludovica Nucci, Rosario Serpico, Gilberto Sammartino, Loredana Capozzi, Antonio Parisi, Marina Di Domenico, Felice Lorusso, Maria Contaldo, Francesco Inchingolo, Gianna Dipalma

**Affiliations:** 1 Department of Interdisciplinary Medicine, University of Medicine Aldo Moro, 70124 Bari, Italy; ad.inchingolo@libero.it (A.D.I.); angeloinchingolo@gmail.com (A.M.I.); luigi.santacroce@uniba.it (L.S.); graziamarinelli@live.it (G.M.); dr.antonio.mancini@gmail.com (A.M.); luigi.vimercati@uniba.it (L.V.); m.e_maggiore@yahoo.it (M.E.M.); mtdoria51@gmail.com (M.T.D.); denisahazballa@gmail.com (D.H.); francesco.inchingolo@uniba.it (F.I.); giannadipalma@tiscali.it (G.D.); 2 Department of Medical and Biological Sciences, University of Udine, Via delle Scienze, 206, 33100 Udine, Italy; 3 Kongresi Elbasanit, Rruga: Aqif Pasha, 3001 Elbasan, Albania; 4 Department of Oral Rehabilitation, Faculty of Dentistry, Iuliu Hațieganu University of Medicine and Pharmacy, 400012 Cluj-Napoca, Romania; 5 Department of Dental Prosthesis, Medical University of Tirana, Rruga e Dibrës, U.M.T., 1001 Tirana, Albania; editxhajanka@yahoo.com; 6 Department of Innovative Technologies in Medicine and Dentistry, University of Chieti-Pescara, 66100 Chieti, Italy; ascarano@unich.it; 7 UOC Maxillo-Facial Surgery and Dentistry, Department of Biomedical, Surgical and Dental Sciences, School of Dentistry, Fondazione IRCCS Ca Granda, Ospedale Maggiore Policlinico, University of Milan, 20100 Milan, Italy; marco.farronato@unimi.it (M.F.); gianluca.tartaglia@unimi.it (G.M.T.); 8 Hospital A.O.S.G. Moscati, Contrada Amoretta, 83100 Avellino, Italy; giovanniellodelia@gmail.com; 9 Multidisciplinary Department of Medical-Surgical and Dental Specialties, University of Campania Luigi Vanvitelli, Via Luigi de Crecchio, 6, 80138 Naples, Italy; ludovica.nucci@unicampania.it (L.N.); rosario.serpico@unicampania.it (R.S.); maria.contaldo@unicampania.it (M.C.); 10 Department of Neuroscience, Reproductive Sciences and Dentistry, University of Naples Federico II, 80131 Naples, Italy; gilberto.sammartino@unina.it; 11 Istituto Zooprofilattico Sperimentale Della Puglia e Della Basilicata, 71121 Foggia, Italy; loredana.capozzi@izspb.it (L.C.); antonio.parisi@izspb.it (A.P.); 12 Department of Precision Medicine, University of Campania Luigi Vanvitelli, 80138 Naples, Italy; marina.didomenico@unicampania.it

**Keywords:** SARS-CoV-2, coronavirus-19, therapy, pandemics, children, pregnant

## Abstract

Background: The SARS-CoV-2 pandemic has involved a severe increase of cases worldwide in a wide range of populations. The aim of the present investigation was to evaluate recent insights about COVID-19 infection in children, infants and pregnant subjects. Methods: a literature overview was performed including clinical trials, in vitro studies, reviews and published guidelines regarding the present paper topic. A descriptive synthesis was performed to evaluate recent insights and the effectiveness of therapies for SARS-CoV-2 infection in children, infants and pregnant subjects. Results: Insufficient data are available regarding the relationship between COVID-19 and the clinical risk of spontaneous abortion and premature foetus death. A decrease in the incidence of COVID-19 could be correlated to a minor expression of ACE2 in childrens’ lungs. At present, a modulation of the dose-effect posology for children and infants is necessary. Conclusions: Pregnant vertical transmission has been hypothesised for SARS-CoV-2 infection. Vaccines are necessary to achieve mass immunity for children and also pregnant subjects.

## 1. Introduction

Since the beginning of 2019, a new coronavirus, SARS-CoV-2, has caused a new disease called the COVID-19 pandemic [1,2,3]. The recent pandemic of coronavirus-2019 (COVID-19) has had an unprecedent impact on adults severely affected by COVID-19 infection [4,5,6,7,8,9,10,11]. The number of children hospitalised for COVID-19 had immediately appeared lower than adults. However, nowadays there are very few references and data reported in the paediatric intensive care unit (PICU) on COVID-19. There is also missing data specifically concerning the transmission of SARS-CoV-2 in schools before the first wave, probably due to their early closure during the beginning of the pandemic in many countries [12,13]. About COVID-19 and the psychological well-being of children and adolescents, data show that mental disorders in children and adolescents increased significantly in the first year of the pandemic, by 25.2% and 20.5%, respectively. Anxiety, depression, uncertainty and loneliness doubled during the pandemic with a prevalence in older adolescents and girls [14,15,16].

At the same time, there was a decrease in the use of drugs, a benefit derived from isolation [17]. The vulnerability linked to emotional-behavioural symptoms (for example, anxiety) of “pandemic adolescents” has led this population to less internalisation, but they engage in behaviours such as binge drinking, self-destructive behaviours, and obsessive use of social media related to uncontrolled eating attitudes [18]. The change in eating habits in children and adolescents associated with less or no motor activity contributed to an increase in obesity and being overweight [19]. A study that looked at the population of 6–14-year olds showed that nearly 30% of boys reported learning difficulties from home.

Sleep disturbances influenced by restriction measures were also recorded: difficulty falling asleep (28%) and a desire to sleep with parents [20]. All the data confirm the difficulty in adapting to quarantine isolation and stress exposure for children in the development phase, and it would be interesting to evaluate it later to evaluate the consequences, and to improve understanding of the duration of symptoms [18,20].

It has been assessed that children and young people aged under 20, often asymptomatic, have a susceptibility to the infection which is about the half that of people aged more than 20. This is according to results issued in *Nature Medicine* which analysed the transmission patterns of COVID-19 according to data coming from six countries, including Italy [21,22].

A multicentre study which has involved 82 health bodies of 25 European countries has confirmed that COVID-19 shows, with a higher frequency, as a slight disease in children, including newborns. The rate of children and adolescents who develop a severe disease and need support in intensive care units is very low and, in these cases, prolonged forced respiratory assistance is required, often for 1 week or more. Death is generally rare [6].

In this study, data reported that individuals who presented a viral coinfection, namely both infected by SARS-CoV-2 and one or more viral agents, had a higher probability of requiring the intensive care unit than the those only infected by SARS-CoV-2. This was important information for the winter period 2020–2021, when the incidence of other viral infections of the respiratory tract, including the RSV virus (Respiratory Syncytial Virus) and influenza were likely to increase, as we had very few data. That was because in Europe the flu season 2019–2020 had already finished before the study concerning the pandemic started, and it was in its early stage [22]. The scientific evidence available indicate that the infection caused by SARS-CoV-2 in paediatric patients expresses with a more positive clinical outcome than the adult. Indeed, children have a death rate which is much lower than the adults, and it is about 0.06% in the 0–15 age group. To date, information issued in the ISS reports four deaths under 9 years old and no deaths in the 10–19 age group [23]. Despite the small number of infected children, asymptomatic or slightly symptomatic paediatric children give rise to concern, as they may transmit SARS-CoV-2 within the community or schools. It is crucial and urgent to understand the role of children in SARS-CoV-2 transmission, above all for concerns about the reopening of schools and contact among several generations [13]. The relationships available up to now show that COVID-19 seems to be rare in children. A slight peak of circulation seems to appear during the winter; the seasonality of coronavirus infections (HCoV) has shown a peak of higher incidence in December-January-February, although HCoV has also been detected during the whole year [24]. HCoV represents a small part of those respiratory infections which require childrens’ hospitalisation and its feature is not very different from other viral respiratory infections [24]. In short, respiratory infections in children due to several coronavirus, act in a similar way and generally have a slight–moderate development with a good clinical course [24,25]. SARS-CoV-2 infections in children have been identified in about 1–2% of cases in China [26]. Although there are cases with a favourable clinical course, also in newborns [27], patients with a critical clinical evolution include 0.6% of babies, but 50% of them are less than 1 year old [28]. Deaths are few and they are almost always associated with comorbidities. For example, in Wuhan a 10-month-old baby with intussusception with multiple organ dysfunction syndrome died 4 weeks after hospitalisation [29]. In several studies conducted on babies Chinese data have shown that of 44,672 confirmed cases of COVID-19 to the 11th of February 2020, 416 (0.9%) were aged between 0 and 10 years old and 549 (1.2%) were aged between 10 and 19 years old [12].

Recent studies on the Italian population have registered all paediatric patients at a rate of 1.8% of all the confirmed cases, with an average age of 11 years old and a minimal prevalence in male patients. Among these, only 13% of patients were hospitalised, while 3.5% of them were transferred to an intensive care unit. The increase of risk is inversely proportional to the age and in cases of comorbidities [30,31]. The strategy of school closures has been adopted all over the world to control the spread of the SARS-CoV-2. However, the real epidemic consequences of this have not been really detected, and it is too early to study the situation. A recent Italian study assessed developments during the transition stage that occurred from the 4th of May to the 13th of September about the spread of COVID-19 infection in paediatric ages in Italy. The purpose was to evaluate the effect of the progressive reopening of all activities before the beginning of the school period [32]. The study reported an increase in the rate of cases diagnosed in children and teenagers from 1.8% (during the block stage) to 8.5% (6197/73,206, during the transition stage). In patients aged less than 18 years, during the first wave, the epidemic peak occurred at the end of March and during the second wave the peak was registered from the last week of August until the half of September. Asymptomatic cases were mostly detected during the second epidemic wave (both for patients aged less than 18 years and other age groups). Since the 4th of May 2020, the date of lockdown suspension, the percentage of contagion was higher for teenagers aged between 13 and 17 years (2558, 41.3%), than the one related to babies aged 7–12 years (1736, 28.0%), 2–6 years (1303, 21.0%), and for the 0–1 year age group (600 9.7%). The patients hospitalised represented 4.8%, among these, babies aged ≤ 1 year were the most affected (16.2%) in the intensive care unit. Among the hospitalised cases, the percentage which required the intensive care unit was 4.3%. Comorbidities were reported in 2.8% of all the cases and in 5.3% of hospitalised patients. The percentage of asymptomatic patients was higher than paucisymptomatic ones (71.2% against the 8.4% of cases of COVID-19), 18.5% of the paediatric population showed a slight infection, with 2% of babies with a severe or critical infection. Among these, newborns aged ≤ 1 year were affected (7.2%). During the first and the second epidemic wave, the infection rate of paediatric cases detected was similar to the lockdown period, but with less hospitalisation, hence with a less severe clinical frame [32]. Control data obtained by the study of several countries revealed that with the opening of schools, the outbreaks that occurred within schools (students and staff) were generally caused by the failure to comply with prevention rules, such as hand-washing, physical distancing and the use of face masks [2,33,34,35,36,37,38,39,40]. The study shows that the spread is higher in secondary schools, while in preschools and among the school staff secondary transmission is almost absent [41]. A study conducted in Germany has shown that the risk of positivity to the SARS-CoV-2 virus in babies was higher in schools attended by babies who lived with a socially disadvantaged background [42]. Babies develop an asymptomatic or paucisymptomatic infection compared to adults, so during the first epidemic stage, as clearly symptomatic subjects were studied, the few sporadic paediatric cases were not detected or were underestimated, by altering the overall data. Indeed, after the *lockdown*, and during the second pandemic wave, the higher diagnostic capacity, the digital infection tracking together with systematic contacts, meant a higher number of cases, also asymptomatic, were detected [32]. Some studies show that symptomatic children, and/or those with a severe clinical frame, are affected by more persistent SARS-CoV-2 in the respiratory ways and in faeces. By showing symptoms which are less frequent in adults, namely a higher quantity of secretion to other respiratory ways as well as gastroenteritis, they encourage the spread of the virus via respiratory and faecal-oral routes [43,44,45,46,47,48,49]. A study by Haiyan Qiu and colleagues, in *The Lancet Infectious Diseases* on 36 patients with COVID-19 (1–6 years), has shown that about half of them (19 patients, 53%) were affected by a slight or asymptomatic disease associated with pneumonia. It is not clear by considering the high rate of asymptomatic infected people, the kind of therapy to adopt, and which children may have to receive antiviral and immunomodulator treatment [50]. These data make us understand that some specific mechanisms which interact in immune and respiratory systems in babies play an important role. This may be the reason why there is a slighter expression of a disease. The pulmonary infiltrates may be a protection during paediatric infection with SARS-CoV-2, as lymphocytes which stimulate the lymphoid structure development associated with bronchi are inducible after a respiratory irritative stimulus [51]. The most frequently observed clinical frames in the paediatric population in a slight or moderate form are: low-grade-fever, dry cough, wheeze, fatigue, nasal congestion, rhinorrhoea, sneezing, abdominal pain, nausea, cephalea, odynophagia, and muscle pain. In the severe form there are: dyspnoea, tachypnoea, severe pneumonia, septic shock, refractory metabolic acidosis, and coagulation disorders [31,50,52]. In fact, children and adolescents that are affected by severe clinical conditions and multisystem COVID-19 pathology, are eligible for hospitalisation and medical treatment protocols.

In paediatric and teen ages, according to data reported in the scientific literature, there seems to be a correlation between SARS-CoV-2 infection and the occurrence of a new rare syndrome, called multisystem acute inflammatory syndrome (MIS-C) with some features of Kawasaki disease associated with other particular clinical expressions for which it is also called “*Kawasaki syndrome*”. It usually occurs after 2–4 weeks and is characterised by a high fever (≥38 °C), signs of toxic shock, encephalopathy, high inflammatory markers, heart and/or gastrointestinal problems, skin rash or bilateral non-purulent conjunctivitis or signs of skin mucus inflammation, with lips and oral mucosa redness, alterations of hands and feet with redness of the hands and feet palms (with or without hard oedema, skin rash and unilateral enlargement of the cervical lymph nodes), even if not present simultaneously, and systemic involvement in general. MIS-C in COVID-19 has some unique features, among which, is the onset in advanced age (cases of teenagers), the prevalence of abdominal symptoms, and more cases with left-ventricular systolic dysfunction. Correlations are still to be defined [53,54,55,56,57,58,59,60,61,62,63,64]. The definitions of the CDC (American Centres for Disease Control and Prevention) and the WHO (World Health Organisation) need real evidence of infection and exposure to SARS-CoV-2, not always possible and hard to report, as in babies infections are usually asymptomatic and the antibody test is not always reliable.

The syndrome is rare (2 in 100,000 people aged less than 21 years), while SARS-CoV-2 infection in people of less than 21 years in the same period report higher numbers (322 in 100,000) [65]. Most patients affected by MIS-C belonged to Black, Hispanic and South Asian people. They had antibodies against SARS-CoV-2 and the virus was detected with a low charge [65,66,67,68]. Heart alterations and the appearance of aneurysms to the coronaries occurred in 10–20% and in some patients the consequences of the disease was the intensive care unit. In severe MIS-C patients with alterations of heart activity there is a high presence of troponin and B natriuretic peptide, and of C-reactive protein, ferritin, lactate dehydrogenase, D-dimers and neutrophils. There is a frequent presence of anaemia, lymphopenia, hypoalbuminemia and coagulation defects. Therapy is based on immunomodulators intravenously, glucocorticoids, antitumor necrosis factor and inhibitors of interleukin-1 or -6. When required, in the most severe cases patients have been supported by the intensive care unit and oxygen. The death rate is 2–4% [66,68].

It is supposed that the immune system is involved in the expression of MIS-C as it only occurs after SARS-CoV-2 infection, after antibody development. There are some studies that show that antibodies may increase the severity of the SARS-CoV-2 infection by triggering inflammation or by mediating damage of the system [69].

In this syndrome as for Kawasaki disease there are genetic alterations in the immune system which put babies at a higher risk by modulating the responses of T and B lymphocytes [70]. Other reasons are that those babies have an innately more active immune response, and they have not been exposed too as much cigarette smoke and atmospheric pollution as adults, so they have healthier respiratory ways with less associated disorders [71,72]. A more important immune response in adults may also justify a dangerous immune response which leads to the acute respiratory distress syndrome [71,73].

A study picked some data, including 157 paediatric patients hospitalised at Wuhan Children’s Hospital, with a diagnosis of severe acute respiratory syndrome caused by coronavirus 2 (SARS-CoV-2) confirmed in the laboratory. Data were picked from the 25th of January to the 18th of April 2020 [74]. Among these 157 COVID-19 paediatric patients, 60 of them (38.2%) detected a slight manifestation, 88 (56.1%) were moderate cases, 6 (3.8%) were severe and 3 (1.9%) were severely sick and needed the intensive care unit. The 148 paediatric patients who showed slight or moderate disease were aged 18 months –10 years and 88 (59.5%) were girls [74]. There were high levels of alanine aminotransferase (ALT), aspartate aminotransferase (AST), creatine kinase activity MB (CK-MB) and lactate dehydrogenase (LDH), and these values were always detected in association with liver and hearth lesions. In slight clinical forms, interleukin-6, tumor necrosis factor α and interferon γ did not present altered values; while the immunosuppressive interleukin-10 level was more increased in moderate cases than slight ones. In slight or moderate cases, the absolute number of lymphocytes (including lymphocytes T and B) was similar; while in moderate cases neutrophils were less than in slight ones. Immunoglobin G and the relationship between neutrophils and lymphocytes were linked in liver and heart damage [69].

Some studies conducted from the 1st of January to the 21st of April 2020 on COVID-19 paediatric patients showed some clinical manifestations: fever (46%) and cough (42%), and diarrhoea, vomit, nasal congestion and fatigue represented 10% in hospitalised paediatric patients. In the laboratory analysis, values reported leukopenia (21%), lymphocytosis (22%), high aspartate aminotransferase (19%), lymphopenia (16%), high alanine aminotransferase (15%), high levels of reactive C-protein (17%), leucocytosis (13%), high D-dimers (12%) and high creatine kinase-MB (5%). The interest of the thorax in the radiographic analysis, unilateral and bilateral, respectively represented 22% in hospitalised paediatric patients. In conclusion, the hospitalised paediatric patients showed slight clinical symptoms, indicators of laboratory tests and features of thorax imaging [75]. In another study of 624 paediatric patients with COVID-19 confirmed by laboratory test, the change in the percentage of leukocytes was only present in 32% of slight paediatric patients, and in these patients creatine kinase MB (CK-MB) was often high. In severe disease, C-reactive protein (CRP), procalcitonin (PCT) and lactate dehydrogenase (LDH) were frequently high. According to these data, the values of leukocytes in babies are not reliable, unlike what has been noted in adults who usually show a leukocyte increase [76]. By considering the data in these cases, systemic inflammation rarely occurs in paediatric patients with COVID-19, in contrast to the lymphopenia and the important and severe inflammatory responses which have been frequently observed in adults with COVID-19. Since the first cases of COVID-19 some cardiovascular events have been reported, among which there are myocarditis, stress cardiomyopathy, myocardial infarction and arrhythmia; through the severity of the critical disease, rather than the lesion of the myocardium from viral particles [77]. Some information on the prevalence of post-COVID-19 complications in young athletes have been obtained [78,79,80]. The use of HMR in athletes after COVID-19 infection, independently on heart symptoms or other heart tests (ECG, echocardiogram and the results of troponin), has an increase of 2.3%, namely 7.4 times the diagnosis based on the symptoms and 2.8 times a screening based on ECG, echocardiogram and troponin [81].

Neurological complications in babies < 18 years affected by COVID-19 are quite frequent: cephalea (4%), anosmia (2%), convulsions (0.7%) and cerebrovascular stroke (0.7%) [82,83].

The pathophysiology of neurological events may be multifactorial: direct viral invasion and replication in the CNS; macro or microvascular circulatory disorders due to vasoconstriction and/or occlusion; nonspecific complications of severe COVID-19, a severe systemic disease such as multisystem inflammatory syndrome in children (MIS-C); and an alteration in the immune system response and autoimmunity [84,85,86].

In this new coronavirus pandemic (SARS-CoV-2) the question is: why are babies less affected than adults? Some main hypotheses must probably be considered [87].

The first hypothesis is dedicated to the enzyme of conversion of angiotensin 2 (ACE2): this receptor is detected on alveolar type 2 cells. A difference in distribution, maturation and functioning of viral receptors is often mentioned as a possible reason for the difference of incidence linked to age [12]. The virus SARS, SARS-CoV-2 and the human coronavirus-NL63 (HCoV-NL63) use the enzyme of conversion of angiotensin 2 (ACE2) as a cellular receptor in human beings. Maybe a minor presence of ACE2 in babies’ lungs influences the clinical expression of COVID-19. This hypothesis should be considered carefully [22,88,89]. It has been observed that ACE2 expression in the lungs of the rat significantly decreases with age. This result may appear noncoherent with a relatively low sensibility of babies to COVID-19. These studies show that ACE2 is involved in the processes of protection of the lung. Severe acute lung lesions triggered by sepsis, acid aspiration, SARS and virus infection of the lethal avian influenza, A H5N1, are protected by ACE2 [88,89]. It has been issued that babies aged less than 1 year represent the group with the highest risk of complications. Infants, indeed, should have a lower ACE2 expression. In these cases, the presence of viral or bacterial coinfections must be taken into consideration and treated promptly. They act as confounders.

The second hypothesis is endothelial damage: it has been stated that age, cardiovascular diseases, diabetes mellitus, and smoke are to considered risk factors for severe COVID-19. Indeed, pre-existent endothelial damage may encourage and increase the inflammatory response from SARS-CoV-2 [87,90,91]. In healthy babies, endothelial damage is basically absent. This may help to avoid the spread of the inflammatory process. The third hypothesis is innate immunity. The first line of defence against SARS-CoV-2 is innate immunity. In order to avoid this, the coronavirus blocks the route of interferon type I to multiply and increase their copies. In conclusion the result of the disease is determined by the balance and combination between the efficacy of the innate and adaptive responses of the host and the virulence and ability of the virus to cancel the immune response of the host [92].

The innate response in babies is stimulated not only by viral infections acquired in the community, but also by the use of vaccines [93,94]. Viral vaccines are mainly administered from the first months of life before the first year and probably this influences the response to SARS-CoV-2 infection. It should also be taken into consideration, the impact of the administration of attenuated RNA vaccines [95]. In this way, the influenza vaccine, which also uses the route of interferon 1, may have an impact on the immune response. This hypothesis on the role of the influenza vaccine immune response, also in the adult population, must be taken into consideration [94,96]. Researchers claim several reasons for the very different progress of COVID-19 among adults and babies. Surely a possible lower maturity and function of the ACE2 receptor in babies if compared to adults causes difficulty in linking the virus and host cells, and finally authors explain the immaturity of the immune system of babies and the possibility to respond in a different way to several pathogenic agents [97]. By starting with this concept, despite having little information on coronavirus, some researchers state that innate immunity plays a fundamental role in the body’s defence against this new virus [96,98]. According to immunology, any virus which breaks the physical barriers of the host, finds a receptor, and a bond site to invade cells, activates some signals and immune responses to avoid and/or slow viral replication. It causes activation of interferon type 1, the activation of natural killer cells and the Toll Like receptors (TLR) responsible for involving several signalling routes [98].

Basically, after the natural defence to the virus, CD8+ T cytotoxic lymphocytes are activated and neutralising antibodies are produced which act above all when the virus is in the extracellular environment by impeding the invasion of many cells, as already observed in the influenza virus [96]. In the case of the new coronavirus, a big RNA positive single stranded virus, TLR7, 8 and 9, together with the adaptor of signal MyD88 stimulate interferon production and activation of NF-κB. This induces some target genes with a cascade stimulation of inflammatory cytokines and the adaptive immunity in this case seems to be unimportant [93,98]. In the ontogenesis of the immune system, the innate immunity is already complete in the first years of life [99].

In adaptive immunity, the cell system activates its functions after the first years of life so that the humoral response improves perfectly after 10 years. A possible explanation for the slight symptoms in babies is surely the perfect innate immunity of this age. Another important result introduced by Dong et al., is that the most severe conditions have occurred in babies aged less than 1 year. This may also be explained by the development of the innate response of this age group, which becomes more effective after the first six months of life [93]. In a study on 32 adults and 47 babies, aged equal to or less than 18 years, it was found that babies mainly produced antibodies directed to the spike protein of SARS-CoV-2, which is useful for the virus to enter cells. Adults produced similar antibodies, but also produced antibodies against the nucleocapsid protein, essential for viral replication and mainly released only when the virus spreads in the whole body. Immune responses in babies seem to be able to eliminate the virus before its spreading [100]. The ability to eliminate the virus in babies may also be related to the fact that they have a great innate immune response from their birth [101]. Babies did not have specific antibodies for the nucleocapsid. This suggests that they are not spreading a living infection, Farber says. The immune response of babies seems to be able to eliminate the virus before its replication.

Another interesting hypothesis is based on the powerful function of neutrophils and their ability to form extracellular nets which encourage system damage and the mortality of COVID-19. If the natural immunity is more dynamic in babies, these big traps may not occur in this age [99]. If we think about the few cases of babies infected, a reason may be that babies travel less and are more socially isolated, so they are less exposed to infect themselves, have a lesser frequency of comorbidity and exposure to smoke compared to adults, and a higher lung regeneration [71,72,102]. Babies and teenagers do not have an old immune system, as observed in elderly individuals, a factor which determines a chronic inflammatory condition [103,104]. Mesenchymal cells have offered great potential. In young patients MSCs have a high rate of proliferation and turnover [105].

In babies and teenagers and young people their differentiation is well preserved. In these patients there are several factors which are involved in different cellular processes, as in the immune response, the proliferation of progenitor cells, angiogenesis, and skeletal muscle metabolism. The presence of long telomeres which leave ACE2 receptors incomplete does not allow the entry of SARS-CoV-2, and the high immune proliferative and stimulating capacity would make the virus invasion difficult. The relationship between length and ageing of telomeres is a multifactorial process [106].

The shortening of each cellular telomere occurs in any division of stem cells. Telomere shortening occurs in a very evident way during acute and chronical inflammation and oxidative stress, as these are conditions which stimulate cell division for tissue repair and immunological responses [107,108].

The regenerative process in babies may represent the key factor which explains their immunity to COVID-19 [105].

## 2. Variants

After the automatic correction of RNA viruses during replication, RNA viruses vary less frequently [109]. The high transmissibility of the new virus variants announced in the beginning of March 2020, raised concerns over several mutations which occurred on the spike (S) glycoprotein [110]. These mutations would modify the interactions of S with the human ACE2 receptor, altering the infection rate, and changing the immune response, and the efficacy of vaccines and monoclonal therapies [111]. Since the beginning of the pandemic, thousands of mutations have occurred. Variants which improve their replication, viral transmission and immune escape increase their frequency [111,112,113].

The Centres for Disease Control and Prevention (CDC) have divided the variants into: variant of interest, variant of concern and variant of high consequence [114].

The first variant of interest (VOI) signalled as B.1.17, also known as 20I/501Y.V1. B.1.1.7, was detected for the first time in December 2020 in the United Kingdom. It was from 40% to 83% more infectious than the wild strain B1 with a higher nasopharyngeal viral load and with a more severe clinical course [115]. The lineage B.1.1.7 has spread in 50 countries, included the United States [115,116].

A study in *The New England Journal of Medicine* has shown an efficacy of the Pfizer-BioNTech vaccine against the English variant of between 87% and 89.5% after at least two weeks after the administration of the two doses [117].

A second study issued in *The Lancet* journal has shown an efficacy higher than 95% against the English variant in fully vaccinated people [118].

The efficacy of vaccines currently available is generally lower against B.1.1.7 [119,120].

The lineage B.1.351 (Beta variant), also known as 20H/501Y.V2 was identified for the first time in South Africa in December 2020, with the first strains in the beginning of October 2020, and since then it has been detected in another 48 countries [121].

Moderna and Pfizer-BioNTech sera have shown a reduced neutralisation of B.1.351 of 12.4 and 10.3 times, respectively [120].

A study in *The New England Journal of Medicine* has shown an efficacy of the Pfizer-BioNTech vaccine of between 72.1% and 75% against the South African variant (hitherto considered the most difficult to fight with the vaccine) after two weeks from the full vaccination.

Novavax and Janssen in a South African report showed a decrease in efficacy of their vaccines against the variant B.1.351 [122].

The administration of two doses of AZD1222, AstraZeneca/Oxford, in South Africa has not protected against slight to moderate COVID-19 because of the variant B.1.351 [122].

The variant P.1, also called 20J/501Y.V3, is a branch of the lineage B.1.28. It was detected in Brazil for the first time, where it has become dominant [123].

The BNT162b2 (Pfizer) and mRNA-1273 vaccines (Moderna) have shown a neutralising action for the variant P.1 lower by 6.7 and 4.5 times, respectively [124].

The variant B.1.61, also known as G/452.V3, was tested in Maharashtra for the first time, in India. Defined as a “double mutant” variant in the sequence encoding the spike protein: E48Q and L452R, it is a mutation which determines a higher transmissibility and infectivity. Three lineage variants have been detected, B.1.617.1 (Kappa), B.1.617.2 (Delta) and B.1.617 [125].

The three lineage variants have a mutation L452R and P618R. The P681R concerns the scission site of furin, which encourages viral entry in lung cells, and this variant is responsible for a higher transmissibility.

The mutations E484Q and L452R determine a stronger bond to the ACE2 receptor, as well as eluding the host immune system more than other variants [126].

On the 10th of May the World Health Organisation (WHO) declared it a “variant of concern”.

Since the 22nd of June 2021 it has spread in 92 countries by becoming from 40% to 60% more contagious than the alpha variant (UK/B.1.1.7) and it could potentially result in being the most infecting variant since the beginning of the pandemic.

In fully vaccinated people, the variant Delta is present in only 3.7% of cases, while in nonvaccinated people this rate rises to 73% [127]. The last studies report that the efficacy of the vaccine BNT162b2 has reduced from 93.4% in the Alpha variant, to 87.9% in Delta after two doses, while the Oxford-AstraZeneca ChAdOx1 vaccine shows a reduction from 66.1% in Alpha to 59.8% with B.1.617.2 [114]. Both Pfizer-BioNTech and Oxford-AstraZeneca have shown their efficacy is just 33% against the Delta symptomatic disease after three weeks from the first dose [125,128].

Both Pfizer-BioNTech and Oxford-AstraZeneca have a reduced risk of hospitalisation against the Delta variant of 96% and 92%, respectively [129].

Antibodies have partially eluded the neutralisation of sera in patients who have been affected by natural infection or immunisation by the administration of the vaccine BNT162b2 and mRNA-1273 against the variant B.1.617, while the sera of people who received one dose of the AstraZeneca/Oxford (ChAdOx) vaccine reported a very low inhibiting response against B.1.617.2 [124,125,129,130,131,132]

At the moment no variant among the ones studied seemed to be more aggressive in any particular age group. All of them have revealed they are mainly transmissible in the same population as the virus which originated COVID-19.

The four variants of concern (VOC) classified by the WHO (World Health Organisation), namely the Alpha variant (former English variant), the Beta variant (former South African variant), the Gamma variant (former Brazilian variant) and the Delta variant (former second Indian variant), all present mutations that make them more transmissible, virulent and/or capable of partially reducing the efficacy of neutralizing antibodies, both those induced by a previous natural infection and those following a SARS-CoV-2 vaccine [133].

The WHO also recognises different variants of interest (VOI) besides the VOC, still poorly studied but able to give rise to new outbreaks, thus representing a possible pandemic threat to keep under control. The latter have also been associated with the Greek alphabet. The WHO epidemiologic update of 22 June 2021, recognised seven variants of interest: Epsilon (B.1.427 and B.1.429); Zeta (P.2); Eta (B.1.525); Theta (P.3); Iota (B.1.526); Kappa (B.1.617.1) and Lambda (C.37) [133,134].

To date, the VOI that mostly raises concern is the Lambda variant or C.37 (code GISAID GR/452Q.V1).

It was detected for the first time in Peru, in December 2020. In May and June 2020, it represented 82% of cases and in June 2021 it was classified a VOI by the WHO.

Believed to be more dangerous than the previous variants, it is suspected to be even more threatening than the Delta variant (which is itself up to 60% more contagious than the already widespread main lineages).

To date, according to the international database GISAID, the Lambda variant is already present in 30 countries. About four thousand cases of the Lambda variant have been registered, most of which are concentrated in South American countries with a peak on 3 May 2021, which began to decrease, slowly at first, and then collapsed starting from 19 July 2021. Currently, data have not changed from March and seem to be continuously declining [135].

Recent studies suggest that the Lambda variant is rather insidious, since it is characterised by a greater contagion chance and a superior resistance to antibodies.

Concerning features of the Lambda variant are due to three mutations present in its spike protein (known as RSYLTPGD246-253N, a 7-amino acid deletion at the N-terminal domain, and L452Q and F490S substitutions). T76I and L452Q, two mutations present in the spike’s receptor binding domain, contribute to make the Lambda variant more infectious.

The RSYLTPGD246-253N mutation (7-amino acid deletion mutation at the N-terminal domain of the Lambda variant spike protein) is responsible for the evasion of neutralising antibodies.

Based upon its greater contagiousness, the L452Q immune escape mutation results are similar to the L452R of the Delta variant. Other mutations are G75V, T76I, del247/253, F490S, D614G and T859N [136].

The Lambda variant results in being the most capable of reducing the efficacy of neutralising antibodies induced by mRNA vaccines (Pfizer and Moderna). It resulted in a 4.6-fold decrease in neutralizing antibodies, even higher than the Beta variant, already known for the capacity of the mutations to evade the immune system [137]. However, all vaccines demonstrated they kept their neutralizing capacity [136,138]. In a recent study conducted on patients who had already contracted the disease, it was possible to notice a few differences in the efficacy of the BNT162b2 or ChAdOx1 nCoV-19 vaccine against the Delta variant versus the Alpha variant after administration of two doses of vaccine. The major differences of vaccine effectiveness were encountered after administration of the first dose. This study suggests the administration of two doses of vaccine, at least among vulnerable populations [129].

In a study conducted in 50 US states until July 2021, data reported that almost all (more than 9/10) COVID-19 hospitalisations and deaths were recorded among nonvaccinated people, or those with only one dose, therefore not fully vaccinated [139].

The last available data show that licensed vaccines (Pfizer-BioNTech, Moderna and Janssen (Johnson & Johnson)) would avoid the severe course of COVID-19 infection and death with both Delta and other variants [140].

The Delta variant is twice as infectious as the original strains circulating at the beginning of the pandemic. A small percentage of people that are fully vaccinated may still be infected. Fully vaccinated people who become infected with the Delta variant are at risk of transmission onto others [141].

WHO declared that the Lambda variant resulted in being more infectious but there are no studies demonstrating its greater aggressiveness [142].

To date, the role of children in the transmission dynamics of these variants has yet to be clarified [143].

In recent months, preliminary results show a great efficacy and tolerability of vaccines against COVID-19 in the age group of twelve to sixteen [144].

## 3. Pregnancy

Mothers affected by COVID-19 should pay great attention to the unborn’s health [145].

The female population, during pregnancy, shows a clinical frame which is similar to the one observed in the common population: with a prevalence of slight cases, few severe cases and reduced cases with a critical frame [146].

Pregnant women who are positive to SARS-CoV-2 are generally asymptomatic in a higher percentage than the general female population infected by the same virus, probably because they are generally younger [147].

Despite there being a low possibility of vertical transmission, a baby whose mother is positive to COVID-19 may have adverse responses, such as foetal distress, preterm birth, respiratory difficulty and death [148].

A study conducted on 20 placentae of mothers positive to SARS-CoV-2 has shown that ten cases in 20 had a foetal vascular malperfusion or vascular thrombosis probably related to the hypercoagulability associated with COVID-19 [66].

Despite to date there being no study that states the relationship between foetal underdevelopment and COVID-19, it is recommended to perform an ultrasound scan 14 days after healing from the acute stage of the disease diagnosed during pregnancy [149,150].

The enzyme for the conversion of angiotensin 2 (ACE 2) is an enzyme on the cellular membrane. It is a component of the renin-angiotensin system (RAS) which interacts with ACE for hydrolysis of the peptide and may modulate blood pressure. ACE 2 hydrolyses angiotensin I (Ang I) to produce angiotensin (1–9) and inactivates the powerful vasoconstrictor Ang II to produce angiotensin-(1–7) [151,152,153].

It is also a bond site of election for SARS-CoV-2, which allows the entry of the virus into the target host cells. ACE 2 is on the walls of many organs: bowel, kidneys, heart, lungs and foetal tissues [154,155].

Foetal ACE 2 plays an important role in the myocardium, lungs and brain growth. In pregnant women ACE 2 is present in a high quantity to balance preeclampsia by modulating angiotensin (1–7) which binds to the receptor Mas, with a vasodilator function and by regulating the homeostasis of fluids [156].

There are studies on Zika, H1N1 and SARS-CoV which show how these viruses produce foetal defects. Little is known on how SARS-CoV-2 is involved in pregnancy. It may encourage the transmission of COVID-19 to the foetus by interacting with foetal ACE 2, leading to morbidity and foetus death [157].

Pregnant women are particularly susceptible to respiratory infections and severe pneumonia, because of the physiological changes due to pregnancy (elevation of diaphragm, higher use of oxygen and oedema of the mucosa of the respiratory tract) and their immunosuppressive condition [158].

A study was conducted on nine pregnant women positive to COVID-19 in the last three months of pregnancy, with pneumonia. The main symptoms were fever and cough, with the main purpose to investigate the possibility of intrauterine transmission of the COVID-19 infection [148].

At the moment of birth, performed with caesarean section, some samples of amniotic fluid, blood from the umbilical cord and neonatal pharyngeal swabs were analysed, in order to test possible intrauterine foetal infection. The choice of caesarean section was made due to the uncertainty on the risk of infection transmission during the delivery from the mother to the child with a vaginal delivery. The results showed that there were no COVID-19 infections transmitted by the mother during the last three months of pregnancy and there is no further evidence of vertical transmission [148,159,160,161,162].

Delivery ways are stated by obstetric provisions, as well as the choice of the anaesthesia required for a caesarean section. As there are no data on the virus transmissibility with a vaginal delivery, this may be taken into consideration by patients with a nonsevere and stable clinical frame [163].

Anyway, the evidence of IgM antibodies against COVID-19 in a single newborn, with high cytokines, suggests that vertical transmission is possible, even if it is not common [164]. IgM antibodies were already present two hours after delivery in a high concentration (IgM antibodies usually do not appear until 3–7 days after infection). IgM antibodies are not transferred to the foetus through the placenta. The high level of IgM antibodies suggests that the newborn has been infected in the uterus. IgG antibodies may be transmitted to the foetus through the placenta and appear later than IgM [164,165].

A recent Chinese study, for the first time, stated that vaginal delivery is safe for COVID-19 positive mothers as all newborns were negative to the SARS-CoV-2 virus [166].

It was also noted that perinatal 2019-nCoV infection may have negative effects on newborns, by causing problems such as foetal distress, premature birth, respiratory difficulties, thrombocytopenia with altered liver functionality, and death [99,167].

**Figure 1:**
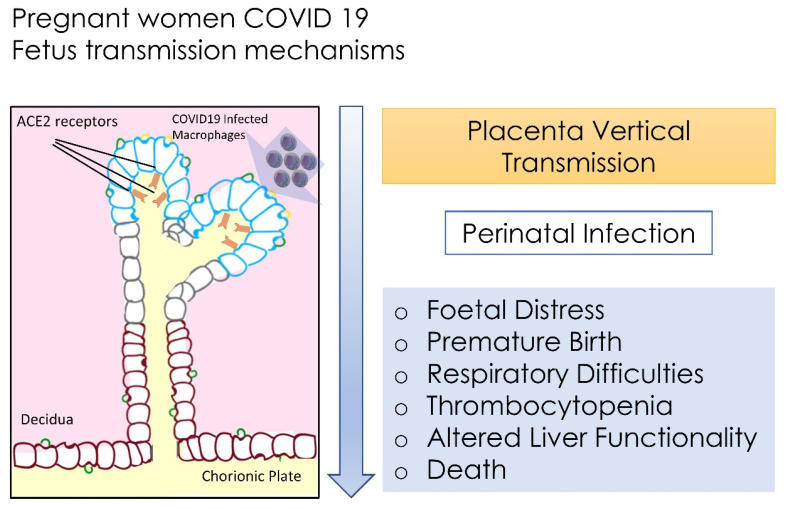
Foetus transmission of SARS-CoV-2 and principal systemic distresses.

There is not sufficient data to show if COVID-19 may cause the risk of spontaneous abortion and premature death. We have little information on congenital infections and teratogenicity of COVID-19 as well as the effects of this disease during the first and second trimester, as there are concerns about the interruption of pregnancy (Figure 1) [163,168].

It would seem that when the infection occurs during the third trimester of pregnancy, it may increase the risk of premature rupture of membranes, preterm birth, foetal tachycardia and foetal distress.

The virus SARS-CoV-2 was not detected in breast milk, but the infection may be transmitted to newborns by close contact, as verified in two cases 36 and 17 h after the birth [169,170].

As many studies confirm the absence of the SARS-CoV-2 virus in the breast milk of infected mothers, there is no indication for separation and interruption of breastfeeding. The use of extracted breast milk is to be considered, when direct breastfeeding is not possible [145,171,172].

Breastfeeding would become hard by hoping that babies from positive mothers may impede possible protection against the virus [173].

The use of PPE by the mother, her continuous disinfection of hands, surfaces and milk containers are the basis of preventing the virus in the newborn [174,175,176,177].

## 4. Psychological Consequences

Domestic isolation during the COVID-19 emergency has determined the occurrence of some behavioural problems and signs of regression in 65% of babies aged less than 6 years, and higher than 71% in those aged more than 6 years (until 18). These are the results of a study conducted in the paediatric hospital Gaslini in Genoa, on the psychological and behavioural effects of the lockdown in babies and teenagers in Italy. The most frequent symptoms were: increased irritability, trouble sleeping and anxiety [178].

The need of social isolation, the decrease in physical activity, and the prolonged stay-at-home, may have the effect of causing or worsening obesity and its comorbidities, with alteration of the intestinal microbiota (dysbiosis) [102,179,180,181,182,183].

The degree of malaise of parents due to the lockdown has influenced the degree of severity of some dysfunctional babies/teens in a negative way [178].

## 5. Therapy

According to a revision of the literature performed by the Scientific Committee SiTIP about the treatments recommended in babies infected by COVID, it states that most babies affected by SARS-CoV-2 infection have a benign clinical course. Hence, it has been established that the choice of pharmacological treatment, different from a support therapy, may be reserved for more severe cases [184].

As there are no sufficient and reassuring studies about the therapy for COVID-19 in babies, the guidelines of COVID-19 treatment have not been drafted and treatments are not standardised [185].

Recently, a group of researchers of paediatric infective diseases of North America stated that only support therapy is recommended for most of cases [117].

The therapy is to administer according to the clinical course (Table 1). Following the WHO recommendations [185] it is possible to divide these [186,187].

These clinical frames are often associated with multisystem inflammatory syndrome (MIS-C) [66]. It infects babies and teenagers including those from 0 to 19 years, characterised by a fever > 3 days associated with a skin rash or bilateral nonpurulent conjunctivitis, or with skin-mucosal inflammation in the oral cavity, hands or feet. There may appear hypotension or shock, alteration in heart function, onset of coagulopathy and gastrointestinal disturbances. There are increases of the erythro-sedimentation speed, C-reactive protein and procalcitonin [190]. The therapeutic approach must be based on the symptom diversity and clinical course. In asymptomatic cases there is no treatment. In moderate and slight cases, antipyretic therapy is recommended.

In severe and critical cases, the therapy varies according to the case conditions. Antipyretic, antiviral, immunomodulator therapies and antibiotics are administered [184].

Aspiration of the airway is performed in cases of obstruction; oxygen therapy, by using a nasal cannula or face masks, hydration and monitoring of diuresis.

## 6. Antipyretic Therapy

Paracetamol for fever >38 °C (10–15 mg/kg each 4–6 h). It is not recommended to use ibuprofen in cases of dehydration, vomiting and diarrhoea, as it may increase the risk of renal failure (Table 2) [191].

## 7. Lactoferrin

Breast milk is rich in lactoferrin, a protein contained in the serum which has several biological properties, such as those of iron metabolism. Lactoferrin links and releases iron. It regulates the immune response and the processes of defence against bacteria, fungi and viruses. Lactoferrin acts on cell receptors and impedes viral anchorage and superficial adhesion to the host cell, thereby inhibiting virus access into the cell [192]. It contributes to the destruction of cell membranes, iron blocking, inhibiting adhesion of pathogenic agents to host cell membranes and the formation of a biofilm, and impedes the growth of many pathogenic agents [193].

In breast milk the peak concentration is detected in colostrum (8 mg/mL); in matured milk there is a lower level (3.5–4 mg/mL), and in exocrine secretions and secondary granules of mature neutrophils, levels are lower still. In the case of infection and/or inflammation, as there are neutrophils, the concentration of lactoferrin increases [193,194,195].

In premature babies, by administering milk higher in concentrations of lactoferrin, the risk of sepsis by intestinal and respiratory means decreases [196,197].

During the first stage of the viral infection process, especially in coronavirus, the virus firstly detects the first sites of cell anchorage. Lactoferrin comes from viral infections as it acts with cell receptors, and is recognised in those of heparan sulphate proteoglycans (HSPG) [198].

Lang et al. have found that lactoferrin inhibits infection because it covers the anchorage sites of the virus provided by HSPG, not allowing the preliminary adhesion between SARS-CoV and the host cells. The blocking of lactoferrin around the process of bonding between the viral spike protein and HSPG is different from that which occurs on the receptor of the enzyme of conversion of angiotensin 2 (ACE2) (Table 3) [199].

Indeed, after the first bond of the virus on the cell surface, the virus detects other receptors which allow the real access to the host cell. Because it interferes with viral anchorage, this process of lactoferrin impedes the following stages, such as the concentration of viruses on the cell surface, and the detection of the bond to specific entry receptors, namely ACE2, which really determine the infection [198,200].

Lang et al. studied these processes on SARS-CoV and not SARS-CoV-2 [198], but SARS-CoV and SARS-CoV-2 have a 72% equal genomic sequence and as the structure of the bond of the receptor is very similar, these data may be valuable [192].

All studies have shown that SARS-CoV-2 is transmitted above all through breath droplets, but it can also infect enterocytes which cause symptoms of gastroenteritis and act as a container. Gastrointestinal symptoms are the main clinical signs in newborns [44,201,202].

Colostrum, the milk of the first months and lactoferrin, thus may create a favourable intestinal microbiota, with anti-inflammatory processes, by stimulating and improving the innate immune defence in newborns [193].

Lactoferrin is considered a powerful regulator of iron homeostasis and of inflammation conditions, as the disturbance of iron homeostasis is related to high levels of IL-6 [194,195].

## 8. Aerosols

Inhalation therapy: a topical steroid and/or bronchodilator in the case of wheeze. The use of nebulisers to avoid aerosols is recommended, as it would increase the transmission of virus, while the use of pressurised suppression with a spacer chamber is recommended. Steroid treatment must not be stopped [203].

Antibiotics must be avoided if there are no signs of bacterial infection. Clinically, a prolonged fever for more than 3 days may indicate the presence of a bacterial infection [204].

It is important to constantly control the vital parameters (Bedside-PEWS) each 8 h or according to the clinical frame [205].

It is important to evaluate the administration of immunomodulators: methylprednisolone or inhibitors of interleukin if available (Anakinra or Tocilizumab). Prednisolone may have immunomodulant activity in the case of ARDS, by decreasing the risk of death. It is recommended in the case of worsening lung function 7 days after symptoms begin, and in the case of an increase in IL-6 and/or D-dimer and/or ferritin and/or C-reactive protein [206].

Even if babies have a much lower incidence of thrombotic complications than adults, the prevention of venous thromboembolism may be made with low molecular weight heparin, although this therapy is not usually provided to babies [207].

Preventive anticoagulant therapy may be considered for newborns and teenagers, where thrombotic events are more frequent [208].

The recommended treatment is with subcutaneous enoxaparin 100–200 U/kg/die, which may be increased to 150–300 U/kg/die in newborns (Table 4).

## 9. Antiviral Treatment

Antiviral therapy for COVID-19 is not necessary for most paediatric patients except for those babies who develop severe or critical diseases.

It is preferable to use antiviral drugs as part of a clinical trial, if available [186].

### 9.1. Lopinavir/Ritonavir

The most recommended antiviral is Lopinavir/Ritonavir. It is used after the first 14 days of life, as a support therapy to other drugs for the human immunodeficiency virus (HIV) [209].

It is available in both an oral suspension and tablets.

Some clinical studies on SARS-COV-2 pneumoniae recommend the early use of Lopinavir/Ritonavir [204].

There is not a sufficient scientific literature on this. The American guidelines on COVID-19 treatment issued in May 2020 suggest using Lopinavir/Ritonavir both for babies and adults, but case-by-case, according to the severity grade of the disease, the need of respiratory support, and by considering the paediatric risk factors for disease progression [186].

Lopinavir/Ritonavir cannot be administered in premature newborns, born before 42 weeks, and in all cases not before their first 14 days of life [186].

The dosing is divided as follows:

### 9.2. Remdesivir

The American Paediatric Infectious Diseases Society for COVID-19 treatment in babies considers Remdesivir as a recommended agent [186].

It has been observed that chloroquine sulphate or hydroxychloroquine sulphate administered together with Remdesivir may contribute to reduce the antiviral activity of Remdesivir [210].

To use Remdesivir, as it is an investigational drug, it is required that procedures for compassionate use be followed (Table 5) [184].

## 10. Hydroxychloriquine

Hydroxychloriquine and chloroquine may perform antiviral activity even if their mechanism is not clear at the moment [211].

In particular, hydroxychloriquine has shown a higher anti-SARS-CoV-2 effect in vitro rather than in vivo. It is not clear if the advantages exceed the risks, above all in babies, for the risk of prolongation of the QT interval and ventricular tachycardia. This risk may also increase if combined with some antibiotics such as the azithromycin (Table 6). Further data are required to assess the possible use of this drug in babies with COVID-19 [212,213].

It is recommended before the use of hydroxychloroquine, to perform an ECG before administration of the drug to exclude a long QT and to dose glucose-6-phosphated dehydrogenase (G6PDH) before use, if there are some risk factors.

## 11. Immunomodulant Therapy

An immunomodulant is administrated in the presence of ARDS (Acute Respiratory Distress Syndrome), or progressive worsening of respiratory activity and MIS (Multisystem Inflammatory Syndrome), an increase of IL-6 and/or D-dimer and/or ferritin and/or C-reactive protein [206].

It is to be used in the case of worsening lung activity after at least 7 days from the beginning of symptoms (Table 7).

## 12. Antibiotic Therapy

The indication for antibiotic therapy is a prolonged fever for more than 3 days, and an indication of bacterial infection [204].

In patients who do not have particular risk factors it is recommended:

− Amoxicillin: 90 mg/kg/die in 3 doses, in the case of possible oral administration.
− Ceftriaxone: 80–100 mg/kg/die, in the case of impossible oral administration. As this drug has the possibility of being administered one time per day, it would reduce exposure risks and transmission for healthcare workers.
− Azithromycin: 15 mg/kg on the first day, then 7.5 mg/kg once a day for another 4 days.

## 13. Tocilizumab

Tocilizumab is a recombinant humanised monoclonal antibody which belongs to immunoglobulin G1 and acts against IL-6 receptors, both soluble and membrane [214].

This drug is already used in the paediatric population for the treatment of moderate and severe rheumatoid arthritis, systemic juvenile idiopathic arthritis (for 1 year), juvenile idiopathic polyarthritis (for 2 years) and severe CAR T cell-induced cytokine release syndrome (T-cell of the chimeric antigen receptor) (for 2 years) [215].

As the alveolar damage in COVID-19 is caused by a cytokine storm (including IL-6), it has been observed that symptoms improve with the use of Tocilizumab [132].

Dosing: vials of Tocilizumab 20 mg/mL:

(1) First infusion: 10–12 mg/kg < 30 kg and 8 mg/kg > 30 kg (max dosing 800 mg, duration of the infusion about 60 min);

(2) Second infusion: 12 h after the first one (according to medical advice, in the case of no response).

It may also be possible to consider a third infusion after 24 h.

At 24 h after the last administration, the plasma dose is assessed for IL-6 and/or D-dimer.

Data from the literature reports that most SARS-CoV-2 infections in babies have a benign trend. Pharmacological treatment, without support therapy, may only be considered for more severe cases.

## 14. Vaccinations

Pregnancy and Breastfeeding.

The WHO, Food and Drug Administration (FDA), Centres for Disease Control and Prevention (CDC), the American College of Obstetricians and Gynaecologists (ACOG), the Canadian Society of Obstetrics and Gynaecology (SOGC), and the Royal College of Obstetricians and Gynaecologists (RCOG) have all confirmed not to exclude from vaccine lists, pregnant and lactating women who present a high risk of exposure to the SARS-CoV-2 virus and/or with health conditions which expose them to a risk of severe maternal and/or foetus/neonatal morbidity after infection. In these specific cases women have to individually evaluate the eventual risks and advantages with their assisting doctors, in order to consciously decide [216,217].

If a vaccinated woman is found to be pregnant immediately after the vaccine, there are no studies which recommend pregnancy interruption.

If a woman is found to be pregnant between the first and second dose of vaccine it would be better to postpone the second dose of vaccine until after the pregnancy, except for high-risk women.

Lactating women may be included in the vaccine campaign without needing to interrupt breastfeeding [217].

Some recent studies report that mRNA COVID-19 vaccines generated a great antibody response in pregnant and breast-feeding women, with immunogenicity and reactogenicity similar to those reported in nonpregnant women. The immune vaccine-related response was higher than the response observed in natural infection. Antibodies have been transmitted to newborns though the placenta and breast milk [130,131]

## 15. Babies and Teenagers

For all four vaccines: Pfizer-BioNTech, Moderna, AstraZeneca and Johnson & Johnson, currently, administration is not provided to babies and teenagers.

European and American paediatric regulations provide two age groups for the trial of all vaccines tested in paediatric ages: from 0–11 years and 12–17 years.

During trial in adults, half of the volunteers receive a placebo, while in paediatric ones there is an initial stage in which more doses are tested to find the ideal one. Nowadays all tested vaccines are used on teenagers. Moderna and Pfizer-BioNTech are now in a more advanced stage.

In teenagers with the placebo administration, the risk is that if they believe they have been vaccinated, they will exhibit more risky behaviours. The American Agency FDA has approved paediatric studies with placebo administration.

At the end of July 2020, the stage three clinical trial of the Pfizer-BioNTech vaccine started in the United States, by recruiting patients of 12 and over. There were 2260 patients aged between 12 and 15 years and 754 participants were aged among 16 and 17 years. A randomised clinical study was performed and controlled with a placebo. Among these, 1131 teenage patients received vaccine and 1129 received a salt placebo. More than half of them were controlled for at least two months after the second dose, made after 3 weeks [218].

The multinationals of Big Pharma announced the 100% efficacy of the vaccine in preventing symptomatic disease, and it triggered a stronger immune response than that observed in young adults, aged 12–15 years.

The FDA reported that the vaccine side effects in the population aged 12–15 years, were similar to those previously reported in the study with participants 16 years and over: pain in the injection site, asthenia, headache, chills or fever of a moderate or severe degree.

In this study 16 cases of COVID-19 were reported, all were among the 978 who received the placebo with no case in the group of 1005 who received the vaccine [218].

On the 11th of May the Food and Drug Administration (FDA) decided to extend the emergency use of the Pfizer-BioNTech mRNA vaccine to 12 years of age. The advantages are higher than the risks even in teenagers.

The Pfizer-BioNTech COVID-19 vaccine must not be administered in patients with a story of severe allergic response to all vaccine components, including anaphylaxis.

At the moment there is no certain data which establishes if the vaccine may impede one-to-one virus infection, or for how much time the vaccine can provide protection [219].

On the 31st of May 2021, the EMA committee for medicinal products for human use (CHMP) also approved an extension of the indication for the Comirnaty, anti-COVID-19 vaccine, in baby patients aged between 12 and 15 years [220].

In March 2021, in a study of stage 1/2/3 Pfizer and BioNTech they administered them first to healthy babies to evaluate if the vaccine produces an immune response against COVID-19 and if it is safe in babies aged between 6 months and 11 years old [218].

The HCMP has stated that the benefits of Comirnaty in this age group are higher, especially in those with conditions which would increase the risk of severe COVID-19 (obesity, heart defects, diabetes and other diseases) [221].

As the number of babies in the study was limited, the trial could not detect rare side effects. Currently the committee that is evaluating the risks for the pharmacovigilance of the EMA is evaluating very rare cases of myocarditis (inflammation of the heart muscle) and pericarditis (inflammation of the membrane surrounding the heart) which occurred after the vaccine with Comirnaty, above all in people aged less than 30. Most of the cases occurred within a week of the vaccination.

Data to the 24th of May 2021 of the Vaccine Adverse Event Reporting System (VAERS) shows that in the period of 30 days after the second dose of the mRNA COVID-19 vaccine, a higher number of myocarditis/pericarditis occurred than expected in young people aged 16–24 years [222].

At the moment, there is no certain data that these cases are due to the vaccine and the EMA is monitoring the situation [222].

Moderna has already completed the trial on teenagers and has now started the stage 2/3 trial of its anti-COVID-19 vaccine on 6750 babies from six months to 11 years in the United States and Canada.

The study of Moderna on babies up to 11 years will be blinded and randomised and divided into two parts. The first part will use and test different dose levels of vaccine in babies. The safety and immune response will be evaluated to choose the right dose to report during the second part of the study. In the second step, paediatric patients will be administered two casual doses of the Moderna vaccine or a placebo, 28 days after the first one [223].

AstraZeneca will simultaneously test. European teenagers of both age groups: 0–11 and 12–17. Tests started in February and involve about 300 babies and teenagers between 6 and 17 years. At the moment any trial is suspended. Further data by HMRA (Healthcare Products Regulatory Agency) will be provided on rare cases of thrombosis and thrombocytopenia that occurred in adults before the administration of other vaccines [224].

The Bambino Gesù Hospital is among those hospitals which adopted the trial: it will treat 660 teenagers aged 12–18 years. The Johnson & Johnson vaccine will be studied on 3500 paediatric patients. As it is a single-dose vaccine, it could be easily administered at school.

The occurrence of variants of coronavirus and their rapid diffusion (Alpha, Beta, Gamma and Delta) has created new concerns about the role of babies in the COVID-19 pandemic. The last data suggest that the variants, including the last one (Delta), spread in a faster way in all age groups [225].

## 16. Conclusions

School reopening is a difficult compromise between epidemiological consequences and the educational needs of babies. Schools have to consider an effective system for contact and isolation tracking to reduce the virus transmission risk. The decision to close (completely or partially) or open schools is studied according to an approach based on the risk, to optimise the benefits from a didactic point of view, wellbeing and health for students, teachers and all school staff and preventing new COVID-19 pandemic waves at the same time. Currently schools seem to be relatively safe, so long as it is possible to adopt all measures, such as face mask wearing, hand washing and ventilate rooms. The militainment of a school education at present depends on the success of the preventive measures adopted by the overall community. Babies and teenager vaccines represent an important step towards reaching mass immunity, as it is a real container for virus circulation. It is necessary to assess the risk–benefit balance. As the risk of the baby to infect is very low, the possibility of having side effects from the vaccine has to be evaluated once we have all the data.

## Figures and Tables

**Table 1:** Summary of the COVID-19 grading and clinical characteristics [188,189].

Clinical Grading	Characteristics
Asymptomatic case	usually detected in the contact track
Slight case	usually is characterised by fever and/or asthenia and/or effects of upper airway with no radiological/sonographic evidence.
Moderate case	characterised by fever and/or fatigue and/or effects on upper airway (cough or slight respiratory distress) and/or inappetence and/or pneumonia reported by radiography of thorax or ultrasonography.
Severe case	characterised by fever together with cough, always associated with one of the following symptoms: oxygen saturation (SpO2) < 92%, cyanosis, intermittent apnoea, severe respiratory distress, high respiratory rate (RR): breaths/minute > 60 > 3 months; >50 3–12 months >> 40 1–5 years; >30 > 5 years) lethargy, convulsions, drowsiness and dehydration.
Critical case	characterised by paediatric acute respiratory distress syndrome (PARDS) alterations of organ function associated with sepsis, septic shock, coma.

**Table 2:** Summary of COVID-19 therapies.

Authors	Drug	Study Design	Experimental Model	Administration Protocol	Results	Test	Control	Subjects/ Specimens	**Study Period**
Venturini et al. 2020	(1) Antipyretic therapy: prefer paracetamol (10–15 mg/kg every 4–6 h). (2) Inhalation therapy. (3) Antiviral. (4) Anticoagulants. (5) Steroids: Methylprednisolone 1–2 mg/kg (max 80 mg) once a day.	Position Paper	-	(1) Lopinavir/ritonavir: 1) 14 days—12 months: 300 mg/75 mg/m^2^ (corresponding to a 3.75 mL/kg) twice a day OR 16/4 mg/kg (corresponding to 0.2 mL/kg) twice a day 2) > 12 months–18 years: if <15 kg: 12/3 mg/kg (corresponding to 0.15 mL/kg) twice a day; if >15 kg: 10/2.5 mg/kg (corresponding to 0.125 mL/kg) twice a day. (2) Hydroxychloroquine: Children: 6 mg/kg (maximum: 400 mg/dose) twice a day on day 1, followed by 3 mg/kg (maximum: 200 mg/dose) twice a day for up to 5 days. (3) Tocilizumab vial 20 mg/mL 3 infusions: First infusion at a dosage of 10–12 mg/kg < 30 kg and 8 mg/kg > 30 kg, (maximum dosage 800 mg, duration of infusion at least 60 min). (4) Antibiotic: Amoxicillin 90 mg/kg/day in 3 doses, in the case of possible oral intake	-	-	-	Children and prepuberal subjects	-
Chiotos et al. 2020	(1) Remdesivir; (2) Hydroxychloroquine; (3) Lopinavir- ritonavir		Multicentre guidelines	Human	(1) Remdesivir: <40 kg: 5 mg/kg IV loading dose on day 1; followed by 2.5 mg/kg IV every 24 h, ≥40 kg: 200 mg IV loading dose on day 1; followed by 100 mg IV every 24 h with a recommended duration up to 10 days, with a 5-day duration favoured for fast responders (5-day vs. 10-day); (2) Hydroxychloroquine: 400 mg PO BID on day 1, followed by 200 mg PO BID for up to 5 days Infants, children, and adolescents 13 mg/kg (maximum: 800 mg) PO followed by 6.5 mg/kg (maximum: 400 mg) PO at 6, 24, and 48 h after initial dose. Lopinavir-ritonavir: Lopinavir 400 mg/ritonavir 100 mg (2 tablets) PO twice daily Neonates aged ≥ 14 days and postmenstrual age ≥ 42 weeks to children aged < 18 years Lopinavir 300 mg/m^2^ (maximum 400 mg/dose) PO twice daily Recommended duration 7–14 days	-	-	-	Children and Infants
Maharaj et al. 2020	(1) Remdesivir; (2) Hydroxychloroquine;	Clinical study simulation	Children of all ages	weight-normalised dosing for children less than 50 kg	in this simulation-based dose-ranging study, paediatric dosing strategies were devised that provided similar exposures between children within different developmental stages and adults. However, the analysis raised concerns regarding hydroxychloroquine use for coronavirus disease 2019 treatment because unbound plasma concentrations were less than those postulated to mediate an antiviral effect.	children less than 50 kg	-	6000 simulated children (birth to 18 years postnatal age) and 1000 simulated adults (age 20–50 years)	1 month

**Table 3:** Summary of COVID-19 lactoferrin adjuvant treatments.

Authors	Drug	Study Design	Experimental Model	Administration Protocol	Results	Test	Control	Subjects/ Specimens	Study Period
Peroni et al. 2020	Natural Lactoferrin	Editorial	-	peak concentration in colostrum (8 mg/mL), lower levels in mature milk (3.5–4 mg/mL)	Lactoferrin, demonstrates potential antiviral effects.	-			
Lang et al. 2011	Lactoferrin	In vitro culture	HEK293E/ACE2-Myc cells, SARS pseudovirus	1, 3 and 10 µM Lactoferrin	Lactoferrin protective role in host defense against SARS-CoV infection blocking the preliminary interaction between SARS-CoV and host cells.	Lactoferrin	Heparin	12 plates	1 h

**Table 4:** Summary of COVID-19 adjuvant aerosol treatments.

Authors	Drug	Study Design	Experimental Model	Administration Protocol	Results	Test	Control	Subjects/Specimens	Study Period
Chen et al. 2020	Interferon-α2b nebulisation, Lopinavir/litonavir; methylprednisolone	Review	Human	Interferon-α2b nebulisation 100,000–200,000 IU/kg for mild cases, and 200,000–400,000 IU/kg for severe cases, two times/day for 5–7 days. Lopinavir/litonavir (200 mg/50 mg) The recommended doses: weight 7–15 kg, 12 mg/3 mg/kg; weight 15–40 kg, 10 mg/2.5 mg/kg; weight > 40 kg, 400 mg/100 mg as adult each time, twice a day for 1–2 weeks; Intravenous methylprednisolone (1–2 mg/kg/day) is recommended for 3–5 days for	-	-	-		-
Parshuram et al. 2020	Patients need tracheostomy, enterostomy feeding device, home oxygen	Multicenter case-control study	children admitted to inpatient units with no limitations on care	patients with chronic conditions (bone marrow or organ transplantation, cardiac disease, severe cerebral palsy), patients with medical devices that might place them at increased risk (tracheostomy, enterostomy feeding device, home oxygen), patients with acute illness (diabetic ketoacidosis, seizures)	PEWS scores for the 12 h ending 1 h before the clinical deterioration event were 8 (5 to 12) in case patients and 2 (1 to 4) in control patients (*p* < 0.0001). The AUCROC curve (95% confidence interval) was 0.87 (0.85 to 0.89). In case patients, mean scores were 5.3 at 20 to 24 h and 8.4 at 0 to 4 h before the event (*p* < 0.0001).	Urgent ICU	Code Blue	2074 subjects	24 h
Dong et al.	IFN-α Lopinavir/ritonavir Ribavirin Chloroquine phosphate Arbido	Editorial	adults and children over 14 days of age	IFN-α inhalation 5 million U or equivalent dose each time, 2 times/day. Lopinavir/ritonavir oral 200 mg/50 mg/capsule, 2 capsules each time, 2 times/day. Ribavirin intravenous 500 mg each time, 2 to 3 times/day in combination with IFN-α or lopinavir/ritonavir. Chloroquine phosphate oral 500 mg (300 mg for chloroquine) each time, 2 times/day. Arbidol 200 mg each time, 3 times/day	Promising results have been achieved efficacy and safety in the treatment of coronavirus disease 2019 (COVID-19)	-	-	-	-

**Table 5:** Summary of the COVID-19 antiviral dosing [184,186].

Antivirals	Dosing
Lopinavir/ritonavir	14 days–12 months: 300 mg/75 mg/m^2^ (corresponding to 3.75 mL/kg) two times per day OR 16/4 mg/kg (corresponding to 0.2 mL/kg) two times per day >12 months–18 years: if <15 kg: 12/3 mg/kg (corresponding to 0.15 mL/kg) two times per day; if >15 kg: 10/2.5 mg/kg (corresponding to 0.125 mL/kg) two times per day
Remdesivir	Babies (< 40 kg): 1st day 5 mg/kg EV (in 30 min), followed by 2.5 mg/kg EV (in 30 min)/die for other 9 days. To date it is not possible to administer this drug before 2 weeks of life and if the weight is <2.5 kg

**Table 6:** Summary of COVID-19 Hydroxychloriquine dosing.

Antivirals	Dosing
Hydroxychloriquine	Babies: 6 mg/kg (max: 400 mg/dose) two times per day on day 1; continue with 3 mg/kg (max: 200 mg/dose) two times per day for a max of 5 days.

**Table 7:** Summary of COVID-19 Immunomodulant dosing.

Antivirals	Dosing
Methylprednisolone	1–2 mg/kg (max 80 mg) one time per day for 2–5 days.
Anakinra	Babies (<40 kg): 1st day 5 mg/kg EV (in 30 min), followed by 2.5 mg/kg EV (in 30 min)/die for another 9 days. 100 mg/0.67 mL endogenously: 8–10 mg/kg/die in 2 or 4 administrations according to the overall dose (until max 100 mg 4 times per day). At 48–72 h after administration, it would be necessary to repeat plasma dosing of IL-6 and/or D-dimer.

## Data Availability

Not applicable.
